# Bilateral Elimination Rule-Based Finite Class Bayesian Inference System for Circular and Linear Walking Prediction

**DOI:** 10.3390/biomimetics9050266

**Published:** 2024-04-27

**Authors:** Wentao Sheng, Tianyu Gao, Keyao Liang, Yumo Wang

**Affiliations:** 1 School of Mechanical Engineering, Jiangsu University of Technology (JSUT), Changzhou 213001, China; shengwt@njust.edu.cn; 2 School of Intelligent Manufacturing, Nanjing University of Science and Technology (NJUST), Nanjing 210094, China; gty@njust.edu.cn; 3 School of Mechatronics Engineering, Harbin Institute of Technology (HIT), Harbin 150001, China

**Keywords:** Bayesian inference system, circular walking, lower limb assistive device, walking activity transition prediction

## Abstract

Objective: The prediction of upcoming circular walking during linear walking is important for the usability and safety of the interaction between a lower limb assistive device and the wearer. This study aims to build a bilateral elimination rule-based finite class Bayesian inference system (BER-FC-BesIS) with the ability to predict the transition between circular walking and linear walking using inertial measurement units. Methods: Bilateral motion data of the human body were used to improve the recognition and prediction accuracy of BER-FC-BesIS. Results: The mean predicted time of BER-FC-BesIS in predicting the left and right lower limbs’ upcoming steady walking activities is 119.32 ± 9.71 ms and 113.75 ± 11.83 ms, respectively. The mean time differences between the predicted time and the real time of BER-FC-BesIS in the left and right lower limbs’ prediction are 14.22 ± 3.74 ms and 13.59 ± 4.92 ms, respectively. The prediction accuracy of BER-FC-BesIS is 93.98%. Conclusion: Upcoming steady walking activities (e.g., linear walking and circular walking) can be accurately predicted by BER-FC-BesIS innovatively. Significance: This study could be helpful and instructional to improve the lower limb assistive devices’ capabilities of walking activity prediction with emphasis on non-linear walking activities in daily living.

## 1. Introduction

Intention is a mental activity that humans and animals use to regulate their activities. In nature, predicting the intentions of other organisms can help prey and predators better adjust their moving trajectories to survive [1]. In human society, predicting the intentions of others is also an essential skill in competitive sports or daily living [2]. Predicting human intentions is also important in the field of robotics. Lower limb assistive devices (e.g., exoskeletons, orthotics, prostheses, etc.) adjust their assist strategies by predicting the walking intention of the wearers, so as to ensure that they can achieve good assist performance on multiple terrains and paths.

Lower limb assistive devices are gradually being applied in assisting human walking [3,4] and fall prevention [5]. In order to make lower limb assistive devices interact with wearers more actively, lower limb assistive devices should be able to provide active assist [6]. Therefore, the movements of lower limb assistive devices should be ahead of wearers’ movements [7]. Lower limb assistive devices adjust their assist strategies before the walking activities of wearers change according to the results of walking intention prediction [7]. Thus, the prediction of wearers’ walking intention is one of the effective ways to realize the active assist of lower limb assistive devices.

Walking intention includes maintaining the current steady walking activity or performing transition walking activity. The walker has to make extra efforts to maintain balance during transition walking activity [8,9]. Upcoming steady walking activity can be predicted according to the recognized transition walking activity and previous steady walking activity. Therefore, the key to predict human walking intention lies in the accurate and fast recognition of steady walking activity and transition walking activity [8]. For this reason, researchers have carried out a lot of research on walking intention recognition algorithms.

In existing studies, sensors used to predict the transition between steady walking activities mainly include EMG electrodes [10,11,12], inertial measurement units (IMUs) [13,14,15], or a combination of them [16,17]. Recognition algorithms using EMG electrodes have the following disadvantages. Firstly, a surface electromyogram (sEMG) is a non-stationary time-varying signal; the amplitude and frequency of the sEMG signal may change due to the fatigue of the operator’s muscles [18]. Secondly, an sEMG signal varies between individuals and is poorly repeatable. Thirdly, an sEMG is highly correlated with human physical characteristics (e.g., corporeity, health, etc.) [19]. Compared with an sEMG, the signal measured by IMUs is more stable and repeatable. Moreover, an IMU does not need to be attached to the skin of wearers. Therefore, an IMU is more convenient for wearing and more suitable for use in daily living [20]. The combination of EMG electrodes and IMUs greatly increases the number of sensors and complexity of the lower limbs’ assistive device. Therefore, more walking intention recognition algorithms use IMUs for data collection.

Roman Stolyarov et al. proposed a novel algorithm for predicting upcoming steady walking activity through estimating leg joints’ translational motion. This algorithm can predict the transitions among linear walking (LW), ramp ascend (RA), ramp descend (RD), stair ascend (SA), and stair descend (SD). The prediction accuracy reaches 96.22% [7]. Ming Liu et al. integrated the environmental information collected by the laser distance meter on the lower limb assistive device and the IMUs on the wearer into the neuromuscular–mechanical fusion-based locomotion mode recognition system. The transitions among LW, RA/RD, and SA/SD can be predicted. The prediction accuracy is over 98%. The decision-making time is more than 500 ms ahead of the real time [21]. Long Yi et al. proposed a PSO-SVM-based online locomotion recognition algorithm, which can predict LW, RA/RD, and SA/SD with an accuracy of 96.00 ± 2.45% [15]. The above algorithms meet the requirements of adjusting the control strategies of lower limb assistive devices in terms of accuracy and speed [22]. However, these algorithms generally ignore the recognition and prediction of LW, clockwise circular walking (CW), and counterclockwise circular walking (CCW) while subjects are walking on non-linear paths or avoiding obstacles. However, CW and CCW account for 8~50% in typical life scenes [23]. In order to improve the applicability of lower limb assistive devices in daily living, the recognition of transition walking activity such as LW→CW (LC), LW→CCW (LCC), CW→LW (CL), or CCW→LW (CCL) is needed. Akiyama et al. found that steady walking activities (e.g., LW, CW, and CCW) and transition walking activities (e.g., LC, LCC, CL, and CCL) could not be recognized only based on unilateral motion data of lower limbs [24]. In order to solve this problem, bilateral motion data of lower limbs were referenced to recognize steady walking activities and transition walking activities in this study. Based on the bilateral motion data of both lower limbs, bilateral elimination rules (BERs) were developed to reduce the number of potential classes before the decision-making, so as to further improve the accuracy of the walking intention prediction.

In order to realize the prediction of upcoming steady walking activity, a bilateral elimination rule-based finite class Bayesian inference system (BER-FC-BesIS) is proposed in this paper. BER-FC-BesIS realizes the prediction of upcoming steady walking activity by recognizing steady walking activity (e.g., LW, CW, and CCW) and transition walking activity (e.g., LC, LCC, CL, and CCL). Major contributions of this paper include (1) BER-FC-BesIS for the prediction of upcoming steady walking activity during non-linear walking on a level ground, (2) bilateral elimination rules for the optimization of prediction accuracy, and (3) the accurate and fast recognition of walking activities and gait events for the optimization of bilateral elimination efficiency. The walking intention prediction method in this paper would be beneficial to optimize lower limb assistive devices’ control strategies while wearers are walking on different terrains especially non-linear paths. This walking intention prediction enhances the human–robot interaction performance of lifting parcels or heavy marching during wearing exoskeletons. Consequently, the interactions between lower limb assistive devices and wearers could be more ergonomic.

The following contents of this paper are organized as follows. Section 2 describes the materials and methods. Section 3 describes the results of the testing experiment. Section 4 contains discussions of the results and future works. Section 5 contains the conclusion.

## 2. Materials and Methods

### 2.1. Subjects and Data Measurements

This paper studies the prediction of the upcoming steady walking activity of healthy adults. Therefore, eight healthy subjects were recruited to this study. The demographic information of subjects is listed in Table 1. The details about the experiments were disclosed to the subjects. All the subjects volunteered and gave their consent to the experiments. All experiments were performed with ethical approval from the Nanjing University of Science and Technology Ethical Review Board.

**Figure 1:**
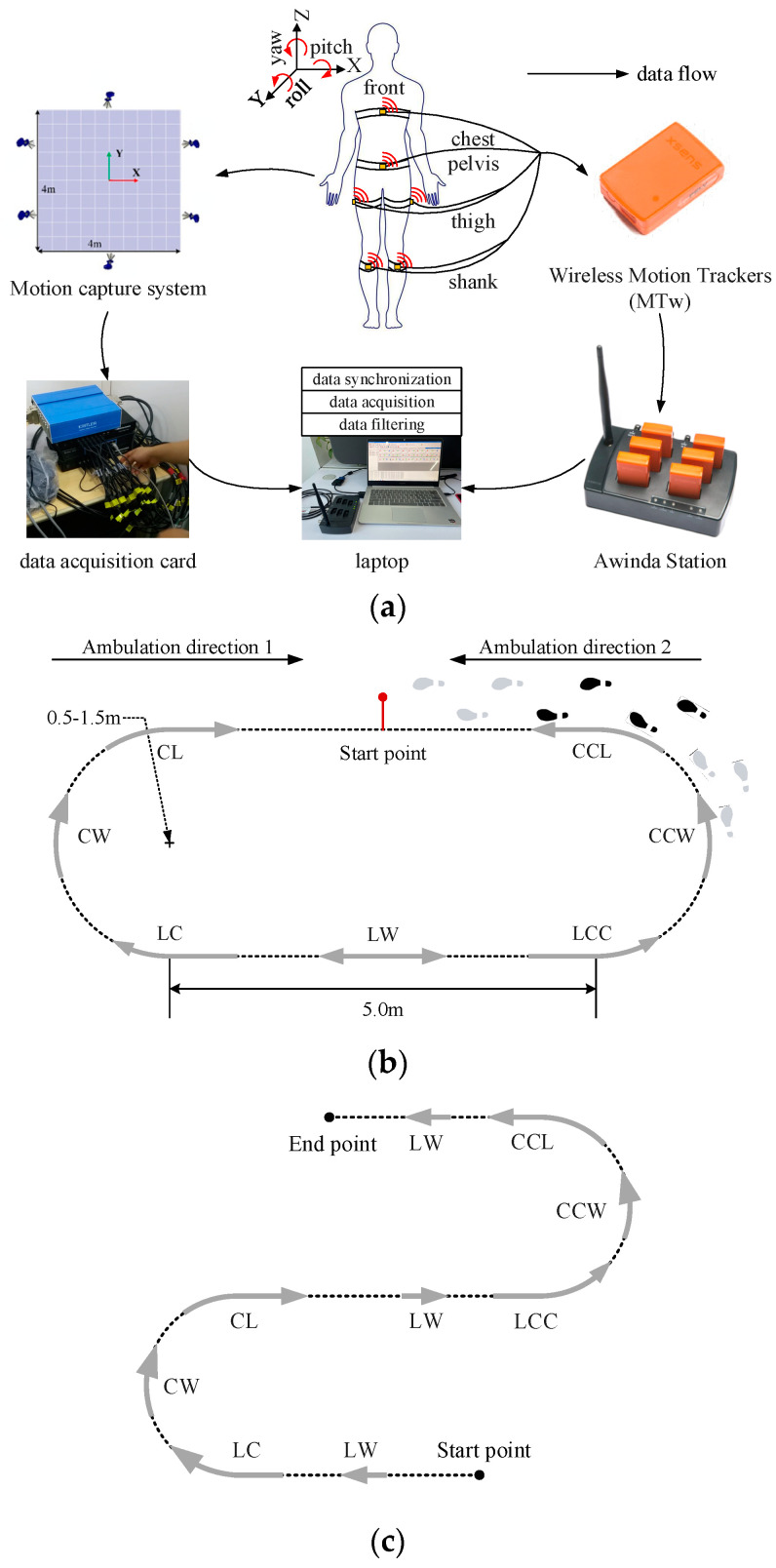
Hardware and venues for data collection experiments. (**a**) MTw is attached to chests, pelvises, thighs, and shanks of subjects, respectively. Data were collected by Awinda Station and Motion capture system at mean time. Collected data were processed on laptop. (**b**) Walking pathway of initial experiments. (**c**) Walking pathway of testing experiments.

The hardware and venues for data collection experiments are shown in Figure 1. As shown in Figure 1a, wireless Motion Trackers (MTw, Xsens Technologies B.V., Enschede, NL, USA) were attached to the subjects’ chests, pelvises, thighs, and shanks for collecting absolute angular velocities. The noise density of MTw is 0.01 deg/s/√Hz. The wireless latency is less than 10 μs. The effectiveness of this sensor attachment in recognizing LW, CW, and CCW as well as gait events within them has been demonstrated [25]. Therefore, this study adopted the same sensor attachment strategy as adopted in [25]. The data collected by MTw were stored in a laptop via the wireless receiver (Awinda Station, Xsens Technologies B.V., Enschede, NL, USA). The motion data of the subjects were also collected synchronously by a six-camera Motion capture system (Motion Analysis Corp., Rohnert Park, CA, USA). The data collected by the Motion capture system were mainly used for labeling the motion data. Both the Motion capture system and MTw set the sampling frequency as 100 Hz. The motion data collected by MTw were stored in a laptop in the form of vectors for the following sequence analysis. As shown in Figure 1b, there are two kinds of walking activities that need to be labeled: steady walking activity and transition walking activity. Steady walking activity includes LW, CW, and CCW. Transition walking activity includes LC, LCC, CL, and CCL. Steady walking activity is persistent. Transition walking activity is a transitional gait cycle between two steady walking activities. As shown in Figure 1b, footprints show how the subject’s walking activity transits from CCW to LW, i.e., CCL, which is represented by the black footprints in Figure 1b. CCW and LW are represented by gray footprints. The definitions of LC, LCC, and CL are the same as that of CCL.

The experiments consist of two stages: the initial experiments and the testing experiments. In all experiments, the subjects walked at their self-selected velocities. The motion data collected in the initial experiments were used to build the likelihood estimation model [25]. The motion data collected in the testing experiments were used to test the performance of FC-BesIS and BER-FC-BesIS.

The walking pathway of the initial experiments is shown in Figure 1b, which consists of straight paths (length: 5 m) and circular paths (radius: 0.5 m, 0.75 m, 1.0 m, and 1.5 m). Each experiment starts when the subject’s toe off (TO) the start point is lifted and ends when the subject’s heel contacts (HC) the start point again. There are two ambulation directions in the initial experiments. The walking activity sequence of ambulation direction 1 is LW→LC→CW→CL→LW→LC→CW→CL→LW. The walking activity sequence of ambulation direction 2 is LW→LCC→CCW→CCL→LW→LCC→CCW→CCL→LW. Ambulation direction 1 and ambulation direction 2 were performed 5 times by each subject, respectively. In order to avoid the effect of vertigo, there was 2 min of resting after each experiment.

### 2.2. Data Processing

**Figure 2:**
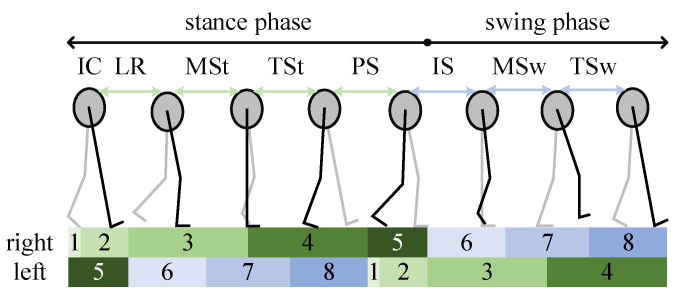
The gait events’ sequences of a bilateral lower limb in a gait cycle. (1: IC, 2: LR, 3: MSt, 4: TSt, 5: PS, 6: IS, 7: MSw, 8: TSw).

The motion data were processed on a laptop (2 GHz CPU, 8 GB RAM). The frequency of subjects’ walking is within 6 Hz, thus 6 Hz second-order Butterworth low-pass filtering is adopted [24]. As shown in Figure 2, each gait cycle is composed of eight gait events: initial contact (IC), loading response (LR), mid-stance (MSt), terminal stance (TSt), pre-swing (PS), initial swing (IS), mid-swing (MSw), and terminal swing (TSw). The data collected by the Motion capture system were used as the references of labeling the motion data collected by the MTw. The threshold method and observation method were both adopted to label the motion data by experienced experts [25].

### 2.3. Motion Feature Extraction

Motion feature extraction is the key to guarantee the accuracy of walking activity recognition. The motion features listed in Table 2 were used as the inputs of BER-FC-BesIS. The effectiveness of these motion features of recognition LW, CW, and CCW has been demonstrated [25].

### 2.4. Finite Class Bayesian Inference System

The prediction of upcoming steady walking activity can be realized by recognizing transition walking activity [13]. As shown in Figure 1c, when the subject wants to transit walking activity from LW to CW, LC will be performed before the walking activity fully transits to CW. Therefore, upcoming steady walking activity can be predicted according to the previous steady walking activity and transition walking activity. FC-BesIS is able to recognize LW, CW, and CCW as well as eight gait events within them. However, only unilateral motion data were referenced for the walking activity and gait event recognition of each lower limb. As demonstrated in [24], unilateral motion data are not enough for classifying LW, CW, CCW, LC, LCC, CL, and CCL. Therefore, this study modified and expanded FC-BesIS to predict upcoming steady walking activity.

The likelihood estimation model was built based on the motion data collected by the MTw in initial experiments. Elimination rule 1 based on the unilateral motion data remained [25]. There are seven potential walking activities and eight potential gait events before elimination rule 1 is performed. The potential classes consist of (walking activity, gait event) pairs. After elimination rule 1 is performed, the number of potential classes is reduced. And a new set of potential classes is obtained. The left lower limb’s potential walking activities, right lower limb’s potential walking activities, left lower limb’s potential gait events, and right lower limb’s potential gait events are all updated by then.

### 2.5. Bilateral Elimination Rules

The recognition accuracy of walking activity and gait events can be improved through reducing the number of potential classes [25]. Therefore, bilateral elimination rules (BERs) were built to further reduce the number of potential classes before decision-making. Elimination rule 2 was built based on the bilateral sequential relationships of walking activities. Elimination rule 3 was built based on the bilateral sequential relationships of gait events.

The bilateral sequential relationships of walking activities during level walking are shown in Table 3. The pseudocodes of elimination rule 2 were built based on the above sequential relationships and are shown in Table 4. After elimination rule 2 is performed, the potential classes of bilateral lower limbs which do not fit the relationships in Table 3 will be eliminated, which means only the walking activity pair consisting of the right lower limb’s potential walking activities and the left lower limb’s potential walking activities within Table 3 can really happen during level walking.

There are also sequential relationships of gait events during level walking [26]. Therefore, the gait events’ sequential relationships of unilateral and bilateral lower limbs were also referenced to build elimination rule 3. A gait cycle can be divided into eight gait events, as shown in Figure 2 [26]. The sequential relationships of these eight gait events are as follows. When the subject’s right heel contacts the ground, the right lower limb’s gait event is IC followed by LR. LR ends when the left toe comes off the ground. The right lower limb’s IC and the left lower limb’s PS start at the same time. The right lower limb’s LR and the left lower limb’s PS end at the same time. After the right lower limb’s LR ends, MSt starts together with the left lower limb’s IS. After the right lower limb’s MSt ends, TSt starts. The right lower limb’s TSt ends together with the left lower limb’s TSw. After the right lower limb’s TSt ends, PS starts together with the left lower limb’s IC. The right lower limb’s PS ends together with the left lower limb’s LR. After the right lower limb’s PS ends, IS starts together with the left lower limb’s MSt. The right lower limb’s TSw ends together with the left lower limb’s TSt [26].

The bilateral sequential relationships of gait events during level walking are shown in Table 5. The pseudocodes of elimination rule 3 are shown in Table 6. After elimination rule 3 is performed, the potential classes of bilateral lower limbs which do not fit the relationships in Table 5 will be eliminated, which means only the gait event pair consisting of the right lower limb’s potential gait events and the left lower limb’s potential gait events within Table 5 can really happen during level walking.

**Figure 3:**
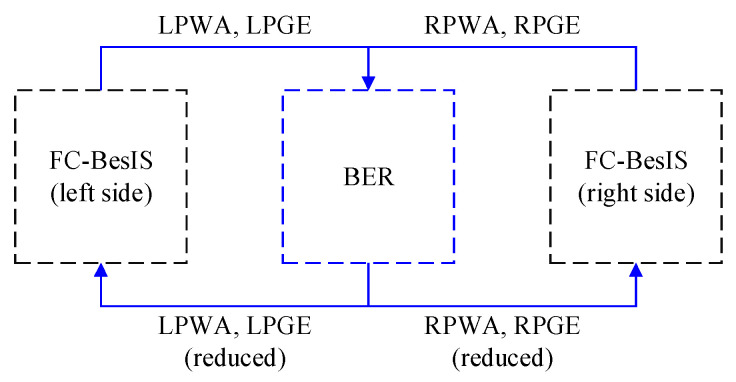
The dataflow between BERs and FC-BesIS. (LPWA: left lower limb’s potential walking activities, LPGE: left lower limb’s potential gait events, RPWA: right lower limb’s potential walking activities, RPGE: right lower limb’s potential gait events).

BERs consist of elimination rule 2 and elimination rule 3. As shown in Figure 3, the left lower limb’s potential walking activities, left lower limb’s potential gait events, right lower limb’s potential walking activities, and right lower limb’s potential gait events will be eliminated by elimination rule 2 and elimination rule 3, respectively. After elimination, the new potential walking activities and gait events will be used for the following recognition processes of BER-FC-BesIS.

### 2.6. Bilateral Elimination Rules-Based Finite Class Bayesian Inference System

The accurate prediction of upcoming walking activity is crucial for the adjustments of lower limb assistive devices’ assist strategies [13]. This requires that BER-FC-BesIS should not only be able to recognize steady walking activity and transition walking activity but also be able to predict the timing when the next steady walking activity starts. When transition walking activity is recognized, the timing of upcoming steady walking activity can be predicted according to the recognized gait event and its proportion within a gait cycle. Therefore, BER-FC-BesIS is designed to recognize not only walking activities (LW, CW, CCW, LC, LCC, CL, and CCL) but also gait events (IC, LR, MSt, TSt, PS, IS, MSw, and TSw). A transition prediction module was also designed for predicting the timing of upcoming steady walking activity. The pseudocodes of the transition prediction module are shown in Table 7. In each gait cycle of normal level walking, the proportion of each gait event is relatively fixed [26]. The proportion of each gait event in a gait cycle is referenced to [26].

**Figure 4:**
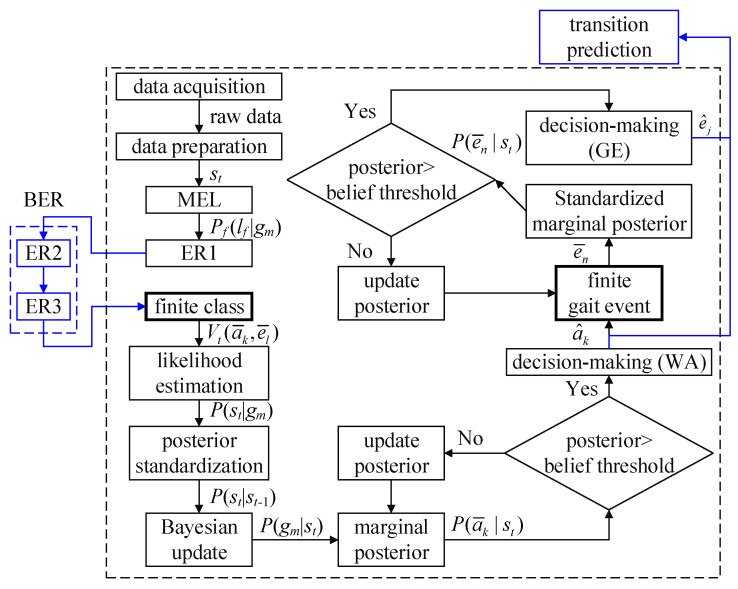
The diagram of BER-FC-BesIS on the right lower limb. The black dashed square is FC-BesIS. The blue lines indicate the expanded module of BER-FC-BesIS. (MEL: likelihood estimation model, ER1: elimination rule 1, ER2: elimination rule 2, ER3: elimination rule 3, WA: module for recognizing walking activities, GE: module for recognizing gait events).

As shown in Figure 4, BER-FC-BesIS is an extension of FC-BesIS (the black dashed square) [25]:

(1) $$P(g_{m}|s_{t})=P(s_{t}|g_{m})P(g_{m}|s_{t-1})/P(s_{t}|s_{t-1})$$

(2) $$P_{uni}(g_{m})=P_{f}(g_{m}|s_{0})=1/M$$

(3) $$P_{f}(l_{f}|g_{m})=h_{f,m}(b)/\sum_{b=1}^{Numbs}h_{f,m}(b)$$

(4) $$P(s_{t}|g_{m})=\frac{1}{F_{features}}\log\prod_{f=1}^{F_{features}}P_{f}(l_{f}|g_{m})$$

(5) $$P(s_{t}|s_{t-1})=\sum_{m=1}^{M}P(s_{t}|g_{m})P(g_{m}|s_{t-1})$$

(6) $$P(a_{i}|s_{t})=\sum_{j=1}^{J}P(a_{i},e_{j}|s_{t})$$

(7) $$P(e_{j}|s_{t})=\sum_{i=1}^{I}P(a_{i},e_{j}|s_{t})$$

where *s_t_* is a vector composed of *F_f_* recorded by MTws at time *t*. *P_f_*(*l_f_*|*g_m_*) is a model of likelihood. $V_{t}({\bar{a}}_{k},{\bar{e}}_{l})$ is the set of the finite class at time *t*. *P*(*s_t_*|*g_m_*) is the likelihood of *g_m_* at time *t*. *P*(*s_t_*|*s_t_*_−1_) is the standardized constant at time *t*. *P*(*g_m_*|*s_t_*) is the posterior probability of *g_m_* at time *t*. $P({\bar{a}}_{k}|s_{t})$ is the standardized marginal posterior probability of ${\bar{a}}_{k}$. ${\bar{a}}_{k}$ is the finite walking activity. ${\hat{a}}_{k}$ is the recognized walking activity. $P({\bar{e}}_{n}|s_{t})$ is the standardized marginal posterior probability of ${\bar{e}}_{n}$. ${\bar{e}}_{n}$ is the finite gait event. ${\hat{e}}_{j}$ is the recognized gait event.

The right lower limb’s BER-FC-BesIS is taken as an example. The processes of BER-FC-BesIS are as follows. Firstly, the likelihood estimation of the collected motion data is performed in a likelihood estimation model module. Secondly, elimination rule 1 is performed according to the results from the likelihood estimation model. Thirdly, the potential classes are further eliminated by elimination rule 2 and elimination rule 3. According to the reduced potential classes, FC-BesIS processes for walking activity and gait event recognition are performed. Once a transition walking activity is recognized by the decision-making (WA) module, the transition prediction module is activated to predict the timing of upcoming steady walking activity.

### 2.7. Statistical Analysis

To evaluate the algorithm performance across all subjects, we conducted a two-way repeated measures analysis of variance (ANOVA) with an α value set to 0.05.

## 3. Results

The performances of FC-BesIS and BER-FC-BesIS on predicting upcoming steady walking activity were evaluated by testing experiments. The results consist of three parts: (1) the walking activity recognition accuracy of FC-BesIS; (2) the gait event recognition accuracy of BER-FC-BesIS; and (3) the walking activity prediction performance of BER-FC-BesIS.

### 3.1. Walking Activity Recognition Accuracy of FC-BesIS

**Figure 5:**
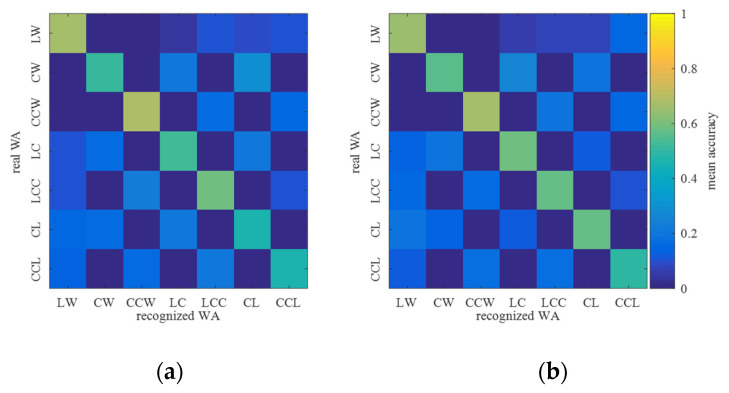
Walking activity confusion matrix of FC-BesIS. (**a**) Confusion matrix of left lower limb’s walking activity recognition. (**b**) Confusion matrix of right lower limb’s walking activity recognition.

FC-BesIS that is based on unilateral motion data was first evaluated in the testing experiments. The mean recognition accuracy of each walking activity was adopted to evaluate the performance of FC-BesIS quantitatively. The walking activity recognition accuracy confusion matrixes of bilateral lower limbs are as shown in Figure 5. Figure 5a is the mean recognition accuracy confusion matrix of the left lower limb’s walking activities. The left lower limb’s mean recognition accuracies of LW, CW, CCW, LC, LCC, CL, and CCL are 67.11%, 50.56%, 68.25%, 51.98%, 58.12%, 46.74%, and 46.06%, respectively. The mean decision time of walking activity recognition is 62.35 ms. Figure 5b is the mean recognition accuracy confusion matrix of the right lower limb’s walking activities. The right lower limb’s mean recognition accuracies of LW, CW, CCW, LC, LCC, CL, and CCL are 65.11%, 55.56%, 66.25%, 57.98%, 57.12%, 56.74%, and 49.06%, respectively. The mean decision time of walking activity recognition is 59.81 ms, across all subjects (*p* < 0.05). The mean recognition accuracy of FC-BesIS is lower than 70%, across all subjects (*p* < 0.05). The low mean recognition accuracy based on unilateral motion data is consistent with the conclusion in [24]. Since the mean recognition accuracy of transition walking activity recognition is low, FC-BesIS is not appropriate for the accurate prediction of upcoming steady walking activity. Therefore, it is reasonable to introduce BER to FC-BesIS for the improvement of mean recognition accuracy.

### 3.2. Gait Event Recognition Performance of BER-FC-BesIS

**Figure 6:**
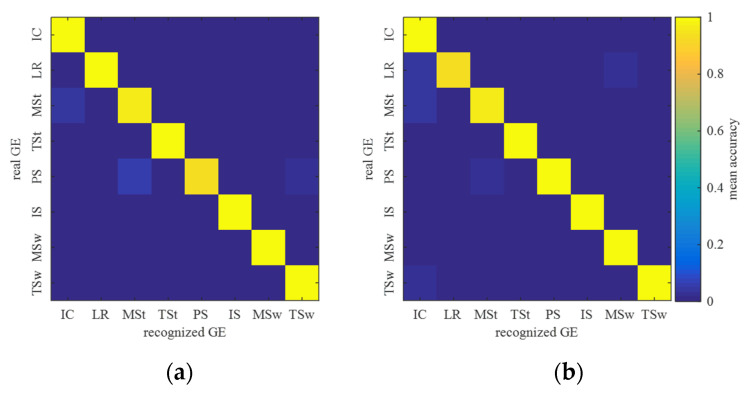
Gait event confusion matrix of BER-FC-BesIS. (**a**) Confusion matrix of left lower limb’s gait event recognition. (**b**) Confusion matrix of right lower limb’s gait event recognition.

The transition prediction module is built based on the sequence and proportion of a gait event in a gait cycle. An accurate recognition of a gait event is the key to ensure the efficiency of a transition prediction module. The gait event recognition accuracy confusion matrixes of bilateral lower limbs are shown in Figure 6. Figure 6a is the mean recognition accuracy confusion matrix of the left lower limb’s gait events. The left lower limb’s mean recognition accuracies of IC, LR, MSt, TSt, PS, IS, MSw, and TSw are 100.00%, 100.00%, 95.67%, 100.00%, 92.67%, 100.00%, 100.00%, and 100.00%. The mean decision time of gait event recognition is 45.98 ms, across all subjects (*p* < 0.05). Figure 6b is the mean recognition accuracy confusion matrix of the right lower limb’s gait events. The right lower limb’s mean recognition accuracies of IC, LR, MSt, TSt, PS, IS, MSw, and TSw are 100.00%, 93.45%, 95.67%, 100.00%, 98.63%, 100.00%, 100.00%, and 98.65%. The mean decision time of gait event recognition is 50.27 ms, across all subjects (*p* < 0.05). The mean recognition accuracies of BER-FC-BesIS on recognizing bilateral lower limbs’ gait events is 98.42%, across all subjects (*p* < 0.05).

### 3.3. Walking Activity Prediction Performance of BER-FC-BesIS

**Figure 7:**
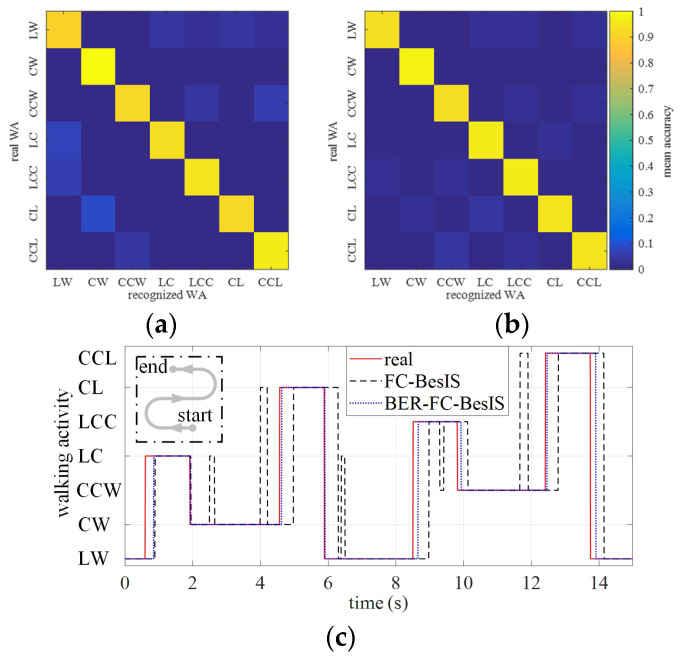
The walking activity confusion matrix of BER-FC-BesIS. (**a**) A confusion matrix of left lower limb walking activities’ recognition. (**b**) A confusion matrix of right lower limb walking activities’ recognition. (**c**) A set of walking activity recognition results of subject 1 within in a test cycle. (red solid line: the real walking activity labeled by an experienced expert according to the motion data of the Motion capture system, black dashed line: the recognized walking activity by FC-BesIS, blue dot dashed line: the recognized walking activity by BER-FC-BesIS).

The walking activity recognition accuracy confusion matrixes of bilateral lower limbs are as shown in Figure 7. Figure 7a is the mean recognition accuracy confusion matrix of the left lower limb’s walking activities. The left lower limb’s mean recognition accuracies of LW, CW, CCW, LC, LCC, CL, and CCL are 89.70%, 99.25%, 91.60%, 92.80%, 94.50%, 92.03%, and 96.06%, respectively. The mean decision time of walking activity recognition is 23.19 ms, across all subjects (*p* < 0.05). Figure 7b is the mean recognition accuracy confusion matrix of the right lower limb’s walking activities. The right lower limb’s mean recognition accuracies of LW, CW, CCW, LC, LCC, CL, and CCL are 92.70%, 98.25%, 93.60%, 95.81%, 96.37%, 94.15%, and 94.02%, respectively. The mean decision time of walking activity recognition is 29.52 ms, across all subjects (*p* < 0.05). Figure 7c shows the recognition results of subject 1 within one testing experiment cycle. The real walking activity, recognized walking activity by FC-BesIS, and recognized walking activity by BER-FC-BesIS are represented by the red line, black dashed line, and blue dot dashed line, respectively. The recognition performance of BER-FC-BesIS is greatly improved over FC-BesIS. The prediction accuracy of BER-FC-BesIS is 93.98%, across all subjects (*p* < 0.05).

**Figure 8:**
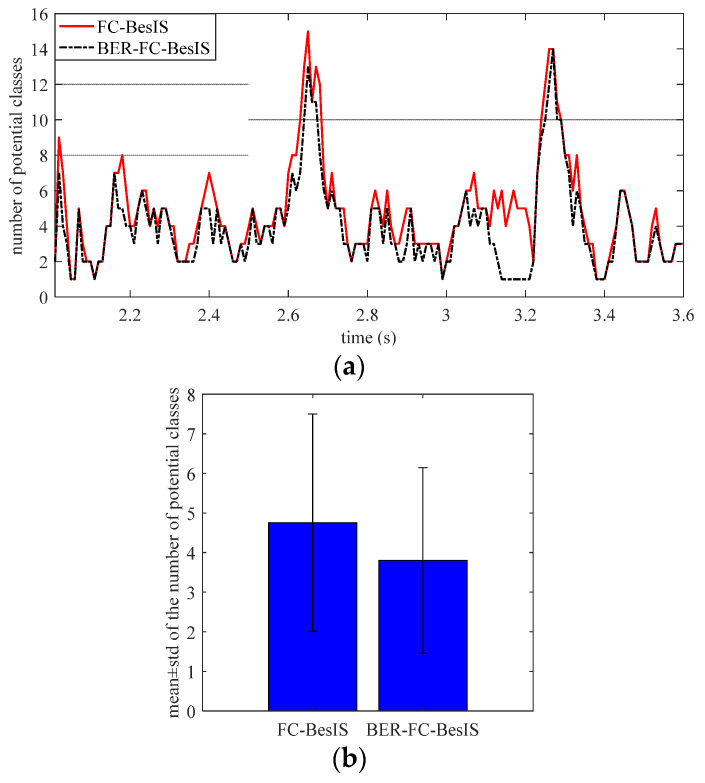
The comparison of potential classes between FC-BesIS and BER-FC-BesIS. (**a**) The number of potential classes before decision-making of FC-BesIS and BER-FC-BesIS. This is a sample of 2–3.6 s in Figure 7. (**b**) The mean ± std of the number of potential classes in FC-BesIS and BER-FC-BesIS.

As shown in Figure 8a, the numbers of potential classes in FC-BesIS and BER-FC-BesIS from 2 to 3.6 s were compared with each other. It is obvious that BER-FC-BesIS eliminated more potential classes than FC-BesIS. As shown in Figure 8b, all through the testing experiments, the mean potential classes of BER-FC-BesIS are 3.95 with a standard deviation of 2.47. The mean potential classes of FC-BesIS are 4.76 with a standard deviation of 2.74. According to bilateral motion data, BER-FC-BesIS reduces more potential classes than FC-BesIS before decision-making. Thus, a higher recognition accuracy as shown in Figure 7a,b is achieved.

Through a transition prediction module, BER-FC-BesIS can predict when the first HC of upcoming steady walking activity is performed by the subject. The time difference between the predicted time and real time is used to quantitatively evaluate the prediction accuracy of BER-FC-BesIS. A positive time difference indicates that the predicted time of upcoming steady walking activity is earlier than the real time of upcoming steady walking activity. A negative time difference indicates that the predicted time of upcoming steady walking activity is later than the real time of upcoming steady walking activity. The mean predicted time and mean time difference are shown in Table 8. The right lower limb’s mean predicted time is 119.32 ms with a standard deviation of 9.71 ms, across all subjects (*p* < 0.05). The left lower limb’s mean predicted time is 113.75 ms with a standard deviation of 11.83 ms, across all subjects (*p* < 0.05). The right lower limb’s mean time difference is 14.22 ms with a standard deviation of 3.74 ms, across all subjects (*p* < 0.05). The left lower limb’s mean time difference is 13.59 ms with a standard deviation of 4.92 ms, across all subjects (*p* < 0.05).

## 4. Discussion

### 4.1. Summary

In this study, BER-FC-BesIS is proposed to predict upcoming steady walking activity (LW, CW, and CCW, etc.) during level walking. The prediction performance of BER-FC-BesIS is quantitively evaluated by testing experiments. The experimental results show that the introduction of BERs greatly improves the accuracy and speed of BER-FC-BesIS in predicting upcoming steady walking activity. To our knowledge, BER-FC-BesIS is the first prediction algorithm that has realized the prediction of the transition between LW, CW, and CCW.

### 4.2. Advantages of BER-FC-BesIS

In state-of-the-art walking activity recognition algorithms, recognition is performed separately by a unilateral lower limb according to unilateral motion data only [27,28,29,30]. Although, steady walking activity such as LC, CW, and CCW can be classified and recognized based on unilateral motion data [24]. In the real world, transition walking activity such as LC, LCC, CL, and CCL are inevitable, and it is hard to distinguish them from LW, CW, and CCW using only unilateral motion data [24]. The mean recognition accuracy of FC-BesIS on recognizing transition walking activities and steady walking activities is lower than 70%. The experimental results show that it is difficult to accurately classify transition walking activities and steady walking activities based on unilateral motion data, which is consistent with the conclusion in [24].

Therefore, in order to improve the recognition accuracy of transition walking activity and steady walking activity, it is necessary to improve the mean recognition accuracy of FC-BesIS. It has been demonstrated that reducing the number of potential classes before decision-making can effectively improve the accuracy and speed of walking activity (LW, CW, and CCW) recognition even only using unilateral motion data [25]. Therefore, it is reasonable to introduce BERs into FC-BesIS. A BER enables the bilateral motion data to be used by unilateral walking activity recognition. Thus, more efficient elimination is achieved with the help of BERs. The prediction accuracy and speed of BER-FC-BesIS meet the requirements of lower limb assistive devices’ control systems [22]. The experimental results demonstrate that the elimination rules based on bilateral sequential relationships of walking activities and gait events can effectively improve the prediction accuracy of upcoming steady walking activity.

### 4.3. Potential Improvements and Future Works

BER-FC-BesIS has realized the accurate prediction of upcoming steady walking activity. The experimental results in Figure 7a,b and Figure 8b show that the accuracy and speed of recognition can be improved by eliminating potential classes before decision-making. By further optimizing and extending the elimination rules, it is expected that BER-FC-BesIS would be able to predict more kinds of upcoming steady walking activities, such as RA/RD and SA/SD.

The recognition and prediction algorithms for walking activities (e.g., LW, RA/RD, SA/SD, etc.) have been studied extensively [28,29,30]. One of the original intentions of this study was to emphasize and verify the prediction of upcoming LW, CW, and CCW possible during level walking. The other intention of this study is to lay the foundation of the following studies in non-linear walking prediction by proposing a prediction algorithm. Therefore, this study did not study and test the performance of BER-FC-BesIS in the recognition and prediction of linear walking activities, such as LW, RA/RD, and SA/SD. However, it can be seen from [31], even an original BesIS is able to accurately recognize walking activities, such as LW, RA/RD, and SA/SD. As an extension of BesIS, BER-FC-BesIS has the potential to recognize and predict linear walking activities, such as LW, RA/RD, and SA/SD. To verify this hypothesis, the performance of BER-FC-BesIS in the recognition and prediction of linear walking activities will be tested in our future works.

## 5. Conclusions

The major contribution of this study is to demonstrate that LW, CW, CCW, LC, LCC, CL, and CCL can not only be recognized but also be predicted. BER-FC-BesIS is proposed in this paper to accurately predict upcoming steady walking activity (e.g., LW, CW, and CCW) by recognizing transition walking activity (e.g., LC, LCC, CL, and CCL). The introduction of bilateral elimination rules greatly improved the prediction performance of BER-FC-BesIS. The testing experiments’ results demonstrate that the mean predicted time and mean time difference in BER-FC-BesIS both meet the requirements of the lower limb assist devices’ control strategies’ adjustments. Furthermore, this study provides a new insight into the prediction of walking activities’ transition with emphasis on non-linear walking.

Future work focuses on the prediction of the transition between LW, RA/RD, SA/SD, and CW/CCW. A larger subject pool with amputees will also be adopted to further test the performance of BER-FC-BesIS.

## Figures and Tables

**Table 1:** Subjects’ demographic information.

Subject	Gender	Age (Years)	Height (cm)	Weight (kg)
1	Male	28	180.0	75.2
2	Male	32	178.2	72.4
3	Male	34	175.5	69.5
4	Male	22	181.3	78.0
5	Male	42	169.2	67.3
6	Female	23	165.0	51.5
7	Female	21	160.3	47.2
8	Female	45	158.4	48.0
Mean [SD]	-	30.9 [9.1]	171.0 [9.0]	63.6 [12.7]

**Table 2:** The motion features for the recognition algorithm.

Features	Signals
1	Pelvis yaw angular velocity
2	Chest yaw angular velocity
3	Left thigh yaw angular velocity
4	Right thigh yaw angular velocity
5	Pelvis roll angular velocity
6	Left shank yaw angular velocity
7	Right shank yaw angular velocity
8	Chest pitch angular velocity
9	Right shank pitch angular velocity
10	Right shank pitch angular velocity
11	Left shank pitch angular velocity
12	Left thigh pitch angular velocity

**Table 3:** The sequential relationship of bilateral walking activities.

Right Lower Limb’s Potential Walking Activities	Left Lower Limb’s Potential Walking Activities
1	1, 4, 5, 6, 7
2	2, 4, 6
3	3, 5, 7
4	1, 2, 4
5	1, 3, 5
6	1, 2, 6
7	1, 3, 7

1: LW, 2: CW, 3: CCW, 4: LC, 5: LCC, 6: CL, 7: CCL.

**Table 4:** Pseudocodes of elimination rule 2 in right side.

After the potential classes have been eliminated by elimination rule 1
IF (right lower limb’s potential walking activity, left lower limb’s potential walking activity) belongs to walking activity pairs in Table 3 THEN DO reserve potential classes with same right lower limb’s potential walking activities
ELSE DO eliminate potential classes with same right lower limb’s potential walking activities
END IF

**Table 5:** The sequential relationship of bilateral gait events.

Right Lower Limb’s Potential Gait Events	Left Lower Limb’s Potential Gait Events
1	5
2	5, 6
3	6, 7
4	7, 8
5	1, 2, 8
6	2, 3
7	3, 4
8	4, 5

1: IC, 2: LR, 3: MSt, 4: TSt, 5: PS, 6: IS, 7: MSw, 8: TSw.

**Table 6:** Pseudocodes of elimination rule 3 in right side.

After the potential classes have been eliminated by ER 2
IF (right lower limb’s potential gait event, left lower limb’s potential gait event) belongs to gait event pair in Table 5 THEN DO reserve potential classes with same right lower limb’s potential gait events
ELSE DO eliminate potential classes with same right lower limb’s potential gait events
END IF

**Table 7:** Pseudocodes of transition prediction module.

After the walking activity and gait event are recognized
DO Calculate MGCT (the mean time of the last three gait cycles, MGCT). IF transition walking activity is recognized THEN IF gait event is IC or LR THEN DO The first HC of the next steady walking activity will occur after 0.9*MGCT ELSE IF gait event is MSt THEN DO The first HC of the next steady walking activity will occur after 0.7*MGCT ELSE IF gait event is TSt THEN DO The first HC of the next steady walking activity will occur after 0.5*MGCT ELSE IF gait event is PSw THEN DO The first HC of the next steady walking activity will occur after 0.4*MGCT ELSE IF gait event is IS THEN DO The first HC of the next steady walking activity will occur after 0.27*MGCT ELSE IF gait event is MSw THEN DO The first HC of the next steady walking activity will occur after 0.13*MGCT ELSE DO The first HC of the next steady walking activity will occur within 0.13*MGCT END IF
ELSE DO The transition prediction module is skipped
END IF

**Table 8:** The mean predicted time and mean time difference.

	MPT ± STD (ms)	MTD (±STD) (ms)
Right	119.32 ± 9.71	14.22 ± 3.74
Left	113.75 ± 11.83	13.59 ± 4.92

MPT: mean predicted time, MTD: mean time difference between predicted time and real time, STD: standard deviation.

## Data Availability

Data are unavailable due to privacy or ethical restrictions.
